# A Narrow pH Range Supports Butanol, Hexanol, and Octanol Production from Syngas in a Continuous Co-culture of *Clostridium ljungdahlii* and *Clostridium kluyveri* with In-Line Product Extraction

**DOI:** 10.3389/fmicb.2016.01773

**Published:** 2016-11-08

**Authors:** Hanno Richter, Bastian Molitor, Martijn Diender, Diana Z. Sousa, Largus T. Angenent

**Affiliations:** ^1^Department for Biological and Environmental Engineering, Cornell UniversityIthaca, NY, USA; ^2^Laboratory of Microbiology, Wageningen UniversityWageningen, Netherlands; ^3^Atkinson Center for a Sustainable Future, Cornell UniversityIthaca, NY, USA

**Keywords:** alcohol, hexanol, octanol, syngas, *Clostridium ljungdahlii*, *Clostridium kluyveri*, co-culture

## Abstract

Carboxydotrophic bacteria (CTB) have received attention due to their ability to synthesize commodity chemicals from producer gas and synthesis gas (syngas). CTB have an important advantage of a high product selectivity compared to chemical catalysts. However, the product spectrum of wild-type CTB is narrow. Our objective was to investigate whether a strategy of combining two wild-type bacterial strains into a single, continuously fed bioprocessing step would be promising to broaden the product spectrum. Here, we have operated a syngas-fermentation process with *Clostridium ljungdahlii* and *Clostridium kluyveri* with in-line product extraction through gas stripping and product condensing within the syngas recirculation line. The main products from *C. ljungdahlii* fermentation at a pH of 6.0 were ethanol and acetate at net volumetric production rates of 65.5 and 431 mmol C·L^−1^·d^−1^, respectively. An estimated 2/3 of total ethanol produced was utilized by *C. kluyveri* to chain elongate with the reverse β-oxidation pathway, resulting in *n*-butyrate and *n*-caproate at net rates of 129 and 70 mmol C·L^−1^·d^−1^, respectively. *C. ljungdahlii* likely reduced the produced carboxylates to their corresponding alcohols with the reductive power from syngas. This resulted in the longer-chain alcohols *n*-butanol, *n*-hexanol, and *n*-octanol at net volumetric production rates of 39.2, 31.7, and 0.045 mmol C·L^−1^·d^−1^, respectively. The continuous production of the longer-chain alcohols occurred only within a narrow pH spectrum of 5.7–6.4 due to the pH discrepancy between the two strains. Regardless whether other wild-type strains could overcome this pH discrepancy, the specificity (mol carbon in product per mol carbon in all other liquid products) for each longer-chain alcohol may never be high in a single bioprocessing step. This, because two bioprocesses compete for intermediates (i.e., carboxylates): (1) chain elongation; and (2) biological reduction. This innate competition resulted in a mixture of *n*-butanol and *n*-hexanol with traces of *n*-octanol.

## Introduction

Depletion of fossil energy carriers and concomitant emissions of greenhouse gases has stimulated research and development of energy systems that are sustainable and carbon neutral. One avenue, besides wind, solar, and hydroelectric power, is the conversion of producer gas into chemicals and fuels (Daniell et al., 2016). Producer gas can come from industrial off gases from the steelmaking industry or from gasification of solid organic waste streams (biomass and municipal waste; Munasinghe and Khanal, 2010; Molitor et al., 2016). Producer gas, which we refer to as syngas, is composed of mainly carbon monoxide (CO), hydrogen (H_2_), and carbon dioxide (CO_2_), with nitrogen, methane, and other compounds at lower concentrations. Recovery of carbon from syngas is a potential strategy to reduce consumption of fossil energy carriers and to reduce greenhouse gas emissions (Dürre, 2016).

Biological conversion of syngas with carboxydotrophic bacteria (CTB) is currently receiving attention because of the technology transfer to industrial scales at steel mills (Liew et al., 2016; Molitor et al., 2016). Specifically, *Clostridium ljungdahlii* and *Clostridium autoethanogenum* produce ethanol using either CO or H_2_ and CO_2_ as substrates (Mock et al., 2015). Bioprocesses with CTB operate at ambient temperatures and pressures, which is an advantage compared to using metal catalysis to convert syngas into chemicals and fuels (Molitor et al., 2016). In addition, CTB are specific in their product portfolio, and have been suggested to tolerate gas contaminants and fluctuating H_2_/CO ratios better than metal catalysts (Dry, 2002; Liew et al., 2013). The spectrum of useful products from wild-type CTB is narrow with ethanol, 2,3-butanediol, and acetate as the only products that can be produced at a promising selectivity (mol carbon in product per mol carbon in substrate). The environmental conditions determine which of these products are being produced based on thermodynamics rather than genetic control between acidogenesis and solventogenesis (Richter et al., 2016). Some CTB, such as *C. carboxidivorans* P7, also produce *n*-butanol and *n*-hexanol from syngas, however, the achieved production rates and selectivities are low (Bruant et al., 2010; Ramió-Pujol et al., 2015).

At least four different biological strategies have already been used to broaden the product spectrum of syngas fermentation: (1) genetic modification in a pure culture, single bioprocessing step; (2) coupling several separate bioprocessing steps; (3) culturing two or more pure culture strains in a single bioprocessing step; and (4) using an undefined mixed culture (reactor microbiome) in a single bioprocessing step. For the first strategy, genetic modification has already resulted in strains that can produce *n*-butyrate (Ueki et al., 2014), *n*-butanol (Köpke et al., 2010), isopropanol (Köpke et al., 2012), and acetone (Banerjee et al., 2014). With the development of reliable and less labor-intense molecular biological tools for CTB, the product spectrum is likely to be further increased (Walker and Köpke, 2015; Huang et al., 2016). The successful demonstration of the functionality of CRISPR/Cas9 systems in relevant CTB is promising to lead to a considerable increase in the number of genetically modified strains (Wang et al., 2015; Xu et al., 2015; Huang et al., 2016).

For the second strategy, Vasudevan et al. (2014) had fed syngas fermentation broth with ethanol and acetate from a pure-culture of *C. ljungdahlii* to an anaerobic reactor microbiome at mildly acidic pH levels. Because of the inhibition of acetoclastic methanogens at that pH, the syngas fermentation products ethanol and acetate were chain elongated to *n*-butyrate and *n*-caproate (Vasudevan et al., 2014). Recently, this strategy was further improved by producing mostly *n*-caprylate (Kucek et al., 2016). The chain elongation mechanism *via* the reverse β-oxidation pathway has been well described in the literature for *Clostridium kluyveri* (Seedorf et al., 2008; Spirito et al., 2014). Chain elongation of the acetate carbon backbone (C2) with ethanol (C2) as an electron donor, energy source, and source of carbon results in *n*-butyrate (C4), *n*-caproate (C6), and *n*-caprylate (C8). We refer to the last two products as medium-chain carboxylates (MCCs). This microbial pathway generates ATP by producing more reduced products than acetate (Hanselmann, 1991; Kleerebezem and Van Loosdrecht, 2010; Agler et al., 2014; Angenent et al., 2016). The reversed order of the set-up for the two bioprocessing steps was also investigated within a biorefinery concept that combined three separate platform technologies - sugar, carboxylate, and syngas platforms (Richter et al., 2013a). Within that concept, syngas fermentation was placed after chain elongation rather than before to reduce non-extracted carboxylates into their corresponding alcohols. Previous work had already described the reduction of carboxylates into their corresponding alcohols by wild-type CTB during syngas fermentation in a single bioprocess (Perez et al., 2013). Finally, two bioprocessing steps in series combined syngas fermentation with aerobic yeast production to convert acetate into malic acid (Oswald et al., 2016) or C16 and C18 lipids (Hu et al., 2016).

For the third strategy, a co-culture of *C. autoethanogenum* and *C. kluyveri* converted syngas into MCCs and longer-chain alcohols in batch fermentations (Diender et al., 2016). This co-culture produced *n*-butanol (14 mmol C·L^−1^·d^−1^), *n*-hexanol (12 mmol C·L^−1^·d^−1^), *n*-butyrate (41.6 mmol C·L^−1^·d^−1^), and *n*-caproate (17.4 mmol C·L^−1^·d^−1^), besides ethanol and acetate. In these batch fermentations, Diender et al. did not observe products with a carbon chain longer than C6. Besides the production of *n*-butyrate and *n*-caproate from chain elongation, Diender et al. observed the reduction of these carboxylates to *n*-butanol and *n*-hexanol. From previous work by Perez et al. (2013), it seems most plausible that *C. autoethanogenum* reduced the elongated products (i.e., carboxylates) from *C. kluyveri* to the corresponding alcohols in a final pathway. The reduction of carboxylates by *C. kluyveri* cannot be ruled out, however, especially since small amounts of *n*-hexanol were found as a fermentation product in pure culture fermentations with *C. kluyveri* (Barker and Taha, 1942; Thauer et al., 1968; Kenealy and Waselefsky, 1985; Weimer and Stevenson, 2012). The co-culture with *C. autoethanogenum* and *C. kluyveri* was only functional in a pH range from 5.5 to 6.5 and an increase in the pH at the end of the fermentation was a limiting factor for the batch fermentation. A well-established *C. autoethanogenum* culture that consumed CO was critical to avoid toxic effects of the CO on *C. kluyveri* when the co-culture was incubated with shaking for better gas-liquid mass-transfer (Diender et al., 2016).

For the fourth strategy, Ganigué et al. (2016) used an acclimated reactor microbiome to produce varying mixtures of *n*-butanol, *n*-hexanol, *n*-butyrate, and *n*-caproate from syngas during batch fermentations. The main products consisted of ethanol and *n*-butanol with only small quantities of products that were longer than C4. There are many similarities in the microbial pathways that are present when compared to the co-culture study of Diender et al. (2016). However, the production rates with the reactor microbiome were approximately 10-fold lower compared to the defined co-culture: *n*-butanol (2.7 mmol C·L^−1^·d^−1^) and *n*-hexanol (1.6 mmol C·L^−1^·d^−1^). Ganigué et al. (2016) found that the pH level in the batch fermenter was of utmost importance for productivity in the single bioprocess step. Unfortunately, a discrepancy in optimum pH levels between ethanol production with CTB and chain elongation with chain-elongating bacteria, such as *C. kluyveri*, was observed. Ethanol production with CTB is optimal at a mildly acidic pH level of 4.5–5.5 (Worden et al., 1991; Mohammadi et al., 2011). For example, ethanol production has been observed at pH 5.3 in a single-stage bioreactor with optimum growth (Mock et al., 2015). On the contrary, chain elongation to *n*-caproate or *n*-caprylate without product extraction is optimal at a neutral pH level of 6.5–7.5 (Grootscholten et al., 2013). Chain elongation was considerably inhibited at mildly acidic conditions due to the high toxicity of undissociated medium-chain carboxylic acids (pKa ~ 4.9) (Spirito et al., 2014).

The third and fourth strategies used batch fermentation. However, a continuous fermentation system with in-line product extraction is advantageous for an industrial-scale biotechnology production platform, because it leads to less downtime within the process due to continuous production. Therefore, our initial objective was to convert the single bioprocess step from a batch system into a continuous system and to add gas stripping to investigate whether we could generate a broader product spectrum for syngas fermentation by integrating two biological processes into a single bioprocessing step, and optimizing production rates by removing toxic end products. Because of the higher production rates of the defined co-culture compared to the undefined mixed culture (Diender et al., 2016; Ganigué et al., 2016), and due to *C. ljungdahlii*'s excellent ethanol production rates (Martin et al., 2015), we chose another co-culture with *C. ljungdahlii* and *C. kluyveri* in a single bioprocessing step. In our co-culture, *C. ljungdahlii* fermented syngas into acetate and ethanol. These products were used as substrates by *C. kluyveri* to chain-elongate them into longer-chain carboxylates. Further, biological reduction of these carboxylates led to the production of longer-chain alcohols. The last step was most likely catalyzed by *C. ljungdahlii*. At a narrow pH range, we observed longer-chain alcohols, consisting of mostly *n*-butanol and *n*-hexanol with some *n*-octanol. We aimed at increasing the production rates compared to the previous batch-fermentations (Diender et al., 2016). However, the production rates and specificities for each alcohol remained suboptimal and further optimization is needed.

## Materials and methods

### Microbial strains and medium composition

*Clostridium ljungdahlii* strain PETC and *Clostridium kluyveri* strain DSM555 were obtained from ATCC (Manassas, VA). For pre-culturing, *C. ljungdahlii* was routinely grown in modified P7 medium in 160 mL serum bottles with a working volume of 10 mL at 35°C under a syngas headspace (Richter et al., 2013b), and *C. kluyveri* was grown in DSMZ52 medium in 160 mL serum bottles with a working volume of 20 or 50 mL at 35°C under strictly anaerobic conditions without shaking. For the reactor study, we used a medium almost identical to a previously described mineral medium with 2x concentrations of all components compared to the described medium (Mock et al., 2015): (1) 60 mL of mineral salt solution (4 g NaCl, 100 g NH_4_Cl, 5 g KCl, 27.23 g KH_2_PO_4_, 13.33 g MgCl_2_ × 6 H_2_O, and 9.8 g CaCl_2_ per L); (2) 20 mL of vitamin solution [10 mg pyridoxine-hydrochloride, 50 mg thiamine, 50 mg riboflavin, 50 mg calcium pantothenate, 50 mg thioctic acid, 50 mg *p*-aminobenzoic acid, 50 mg nicotinic acid, 50 mg vitamin B_12_, 20 mg biotin, 20 mg folic acid, and 10 mg Mesna (Mercaptoethanesulfonic acid sodium salt) per L]; and (3) 20 mL of trace element solution [2 g NTA (nitrilotriacetic acid), 25.2 mg MgCl_2_, 2.84 g (NH_4_)_2_Fe(SO_4_)_2_, 47.6 mg CoCl_2_, 200 mg ZnSO_4_ × 7 H_2_O, 119 mg NiCl_2_ × 6 H_2_O, 48.4 mg Na_2_MoO_4_, 34.6 mg Na_2_SeO_3_, and 66 mg Na_2_WO_4_ × 2 H_2_O per L]. We added 5 to 20 mL of 100 × diluted antifoam 204 (Sigma Aldrich, St. Louis, MO) per L medium depending on the density of the bacterial culture in the reactor. We also added 1 mM final concentration of cysteine after sparging the medium with N_2_ gas to obtain anaerobic conditions. Finally, we added 0.8 mL sodium sulfide per h from an anaerobic 100 mM stock solution directly to the 1 L reactor to continuously supply H_2_S as an additional sulfur source. This resulted in an apparent H_2_S concentration of 1 mM at a media flow rate of 80 mL·h^−1^ (until hour 1150), and 2 mM after we reduced the media flow rate to 40 mL·h^−1^ on hour 1150.

### Reactor and condenser setup

**Figure 1:**
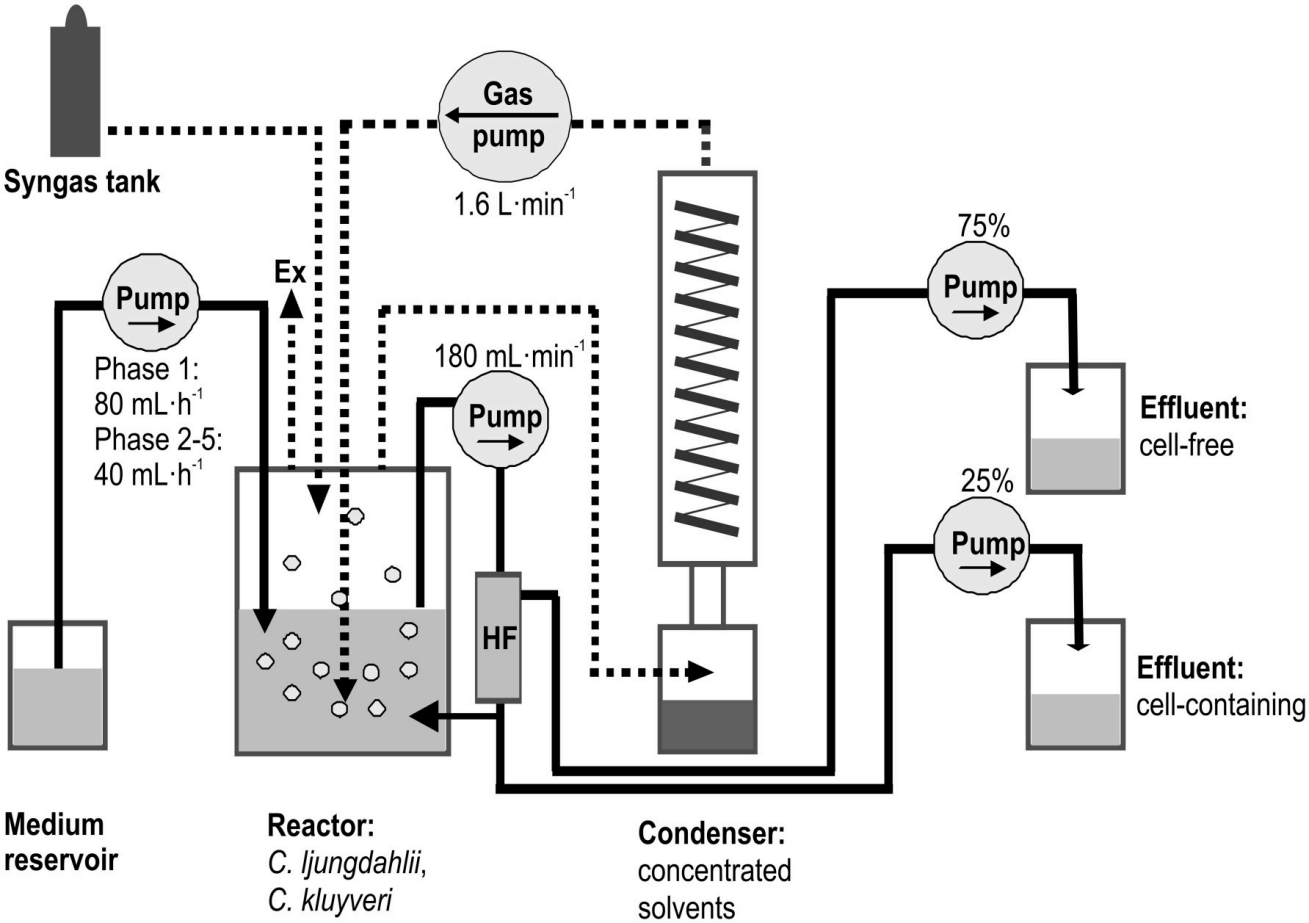
**Schematic view of the reactor**. We operated a single-stage reactor with a co-culture of *C. ljungdahlii* and *C. kluyveri* that was fed continuously from a medium reservoir at a dilution rate of 80 mL·h^−1^ (Phase 1), and 40 mL·h^−1^ (Phase 2–5). The reactor was fed continuously with syngas (60% CO, 35% H_2_, and 5% CO_2_) into the headspace. The gas was recirculated through a gas recycling loop into the reactor broth. A glass condenser unit was installed into the gas recycling loop to extract volatile compounds through gas stripping and condensation. The reactor broth was recirculated over a hollow fiber module (HF). 25% of the continuous reactor effluent consisted of cell-containing broth, while 75% of the reactor effluent was cell-free broth. Ex, exhaust gas outlet.

One 2 L Biostat M chemostat (Braun, Allentown, PA) with a 1 L working volume was used for the reactor experiment (Figure 1). The reactor was initially agitated at 200 rpm (until hour 475), which was then increased to 400 rpm. The temperature was controlled at 37°C by a water jacket. The pH in the reactor was initially only controlled by addition of 2 M KOH (until hour 1510), and then also controlled by addition of 2 M acetic acid (after hour 1510). The reactor was continuously supplied with a synthetic syngas mixture consisting of 60% (vol/vol) CO, 35% H_2_, and 5% CO_2_ (Airgas East, Ithaca, NY) at a gas flow rate of 30–80 mL·min^−1^ (the gas flow rate was adjusted, before gas supply became limiting) into the headspace of the reactor. The gas composition resembles the composition of syngas that is derived from lignocellulosic biomass with gasification such as with plasma gasification. A real producer gas should be tested before full-scale implementation to understand the effect of impurities on the co-culture behavior (Molitor et al., 2016). In addition, the gas was recycled at a gas flow rate of 1.6 L·min^−1^ (Figure 1). The recycled gas was sparged into the reactor broth through a microbubble-generating sparger (MoreFlavor, Concord, CA, USA) made of stainless steel with a pore size of 0.5 μm. We did not determine K_*L*_a-values in this study, but with the high rate of gas recycling (1.6 L·min^−1^) and an agitation speed of 400 rpm, K_*L*_a-values for gas-liquid mass transfer probably exceeded values that we had previously achieved (i.e., 373/h) (Martin et al., 2015), and it is unlikely that mass transfer was a limiting factor in this study. The liquid reactor broth was recirculated through a Cellflo polyethersulfone hollow fiber module with 500 cm^2^ membrane surface area and 0.2 μm pore size (C22E-011-01N, Spectrum Laboratories, Inc., Rancho Dominguez, CA) at a flow rate of 180 mL min^−1^. The media feed rate was initially 80 mL·h^−1^ (until hour 1150), which was then reduced to 40 mL·h^−1^ (Figure 1). The peristaltic eight-channel media pump (ColeParmer, Court Vernon Hills, IL) for continuous operation was set-up in a way that 75% of the effluent consisted of cell-free filtrate coming from the cell-recycling module, and 25% of the effluent was cell-containing reactor broth (to remove dead cell material from the reactor). With respect to the retained cells, this setup resulted in a rate of cell withdrawal (bleed rate) of 1/4 of the applied dilution rate. We cannot easily determine the actual specific growth rates for the two strains. However, during steady-state conditions the growth rates should be identical to the bleed rate, and therefore, 1/4 of the media feed rate (i.e., 1/4·0.08 or 1/4·0.04·h^−1^), respectively. A reflux condenser made of pyrex glass was integrated into the gas recycling line to extract volatile products from the reactor broth by gas stripping and subsequent condensation. The condenser was kept at 1°C by a cooling water bath (Neslab RTE-111, Marshall Scientific, Hampton, NY).

### Analysis

Samples were taken daily from the reactor and from the condensate (*N* = 1). The optical density (OD) was measured at 600 nm using a spectrophotometer (Milton Roy Spectronic 1201, Houston, TX). External pH measurements were performed with a pH probe (Orion Star, A329, Thermo Scientific, Waltham, MA) to confirm and adjust the internal reactor pH control. Ethanol and acetate concentrations were obtained with an HPLC (Shimadzu Prominence UFLC, Shimadzu, Kyoto, Japan), which was equipped with an Aminex HPX-87H analytical column (Bio-Rad, Hercules, CA) at 65°C using 5 mM sulfuric acid in water at a flow rate of 0.6 mL·min^−1^ as eluent. Concentrations of carboxylic acids (C4–C8) were measured using a gas chromatograph as previously described (Perez et al., 2013). Concentrations of higher alcohols (C4–C8) were measured using a gas chromatograph as previously described (Perez et al., 2013), with an initial temperature hold for 3 min at 100°C and a temperature ramp of 40°C·min^−1^ to 220°C, where the temperature was held for 5 min.

Volumetric gas flow rates were measured with a custom-made in-line volumetric flow meter for the inlet gas, and a bubble flow meter for the outlet gas (Richter et al., 2013b). The gas pressure was measured using a digital pressure gauge (ColeParmer, Court Vernon Hills, IL) at the gas inlet and outlet of the reactor. Concentrations of CO, H_2_, and CO_2_ were determined *via* gas chromatography as previously described (Perez et al., 2013). A phase-contrast microscope (Nikon Labophot, Nikon, Melville, NY) was used daily to monitor cell morphology. The volume of reactor effluent (cell-containing and cell-free) was measured to adjust the media flow rate when necessary. The reservoir bottles of the 2 M KOH and 2 M acetic acid solutions were weighed to determine the amount of consumed base and acid.

**Figure 2:**
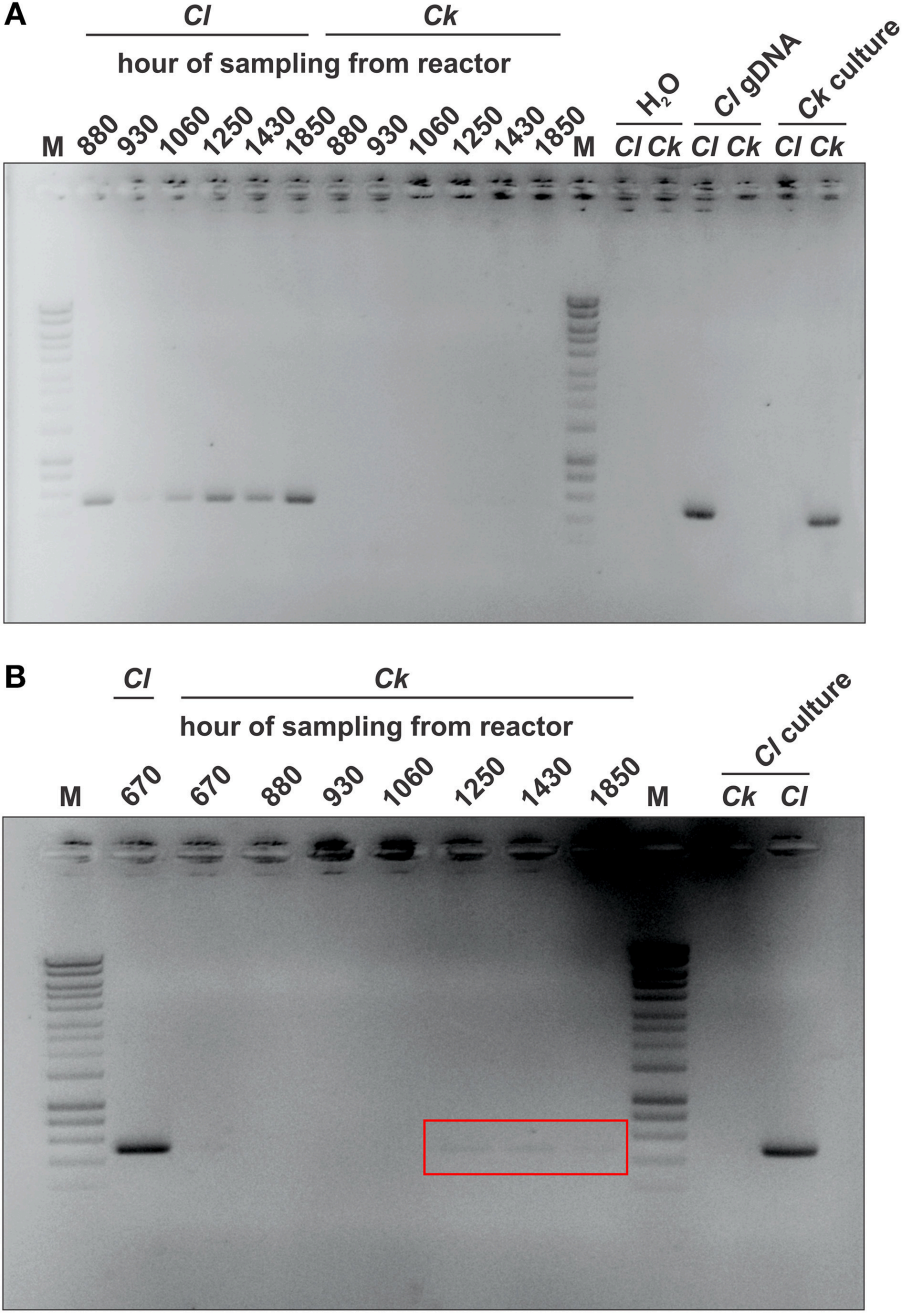
**Qualitative control PCRs for the detection of ***C. ljungdahlii*** and ***C. kluyveri*** in the reactor**. We used specific primer pairs for the detection of either *C. ljungdahlii* (*Cl*) or *C. kluyveri* (*Ck*) in the reactor during the operating period. As a negative control, we used MilliQ water (H_2_O). Primer pairs were tested for cross reactions by using *C. ljungdahlii* genomic DNA (*Cl* gDNA) or a pure culture of *C. ljungdahlii* (*Cl* culture) or *C. kluyveri* (*Ck* culture) as template for each of the primer pairs. **(A)** Each pocket of the agarose gel was loaded with 10 μL of a 50 μL PCR reaction. **(B)** Each pocket was loaded with 40 μL of a 50 μL PCR reaction. Because only faint bands were detected for the *C. kluyveri* specific PCR, visible bands are highlighted with a red box. M, DNA marker (HyperLadder 1kb, Bioline, Singapore).

Non-quantitative, strain-specific control PCRs were conducted to confirm the presence of both microbes in the reactor broth. EconoTaq PLUS GREEN 2X Master Mix (Lucigen, Middleton, WI) was used according to the manufacturer's recommendations in 50 μL reaction volume. Primers specific for *C. ljungdahlii* (forward: 5′-AGTGCAGCGTATTCGTAAGG-3′; reverse: 5′-TAATGAGCCACGTCGTGTTG-3′; locus: 2172133-2172637), and for *C. kluyveri* (forward: 5′-CAAGCCTGGTAGTTGATACG-3′; reverse: 5′-TTAAAGGCCCTCTGTACTCC-3′; locus: 1822201–1822690) were used. MilliQ water, *C. ljungdahlii* genomic DNA, as well as pure cultures of *C. ljungdahlii* and *C. kluyveri* were used to demonstrate the strain-specificity (no cross reactions within the two species) for the primer pairs (Figure 2A). For sample preparation a sterile toothpick was used to transfer some biomass from a reactor cell pellet into 100 μL of DNA-free water. The sample was boiled for 10 min before 1 μL was used as template in the 50 μL PCR reaction.

## Results

### *n*-octanol (C8) observed for first time in a syngas fermentation system

**Figure 3:**
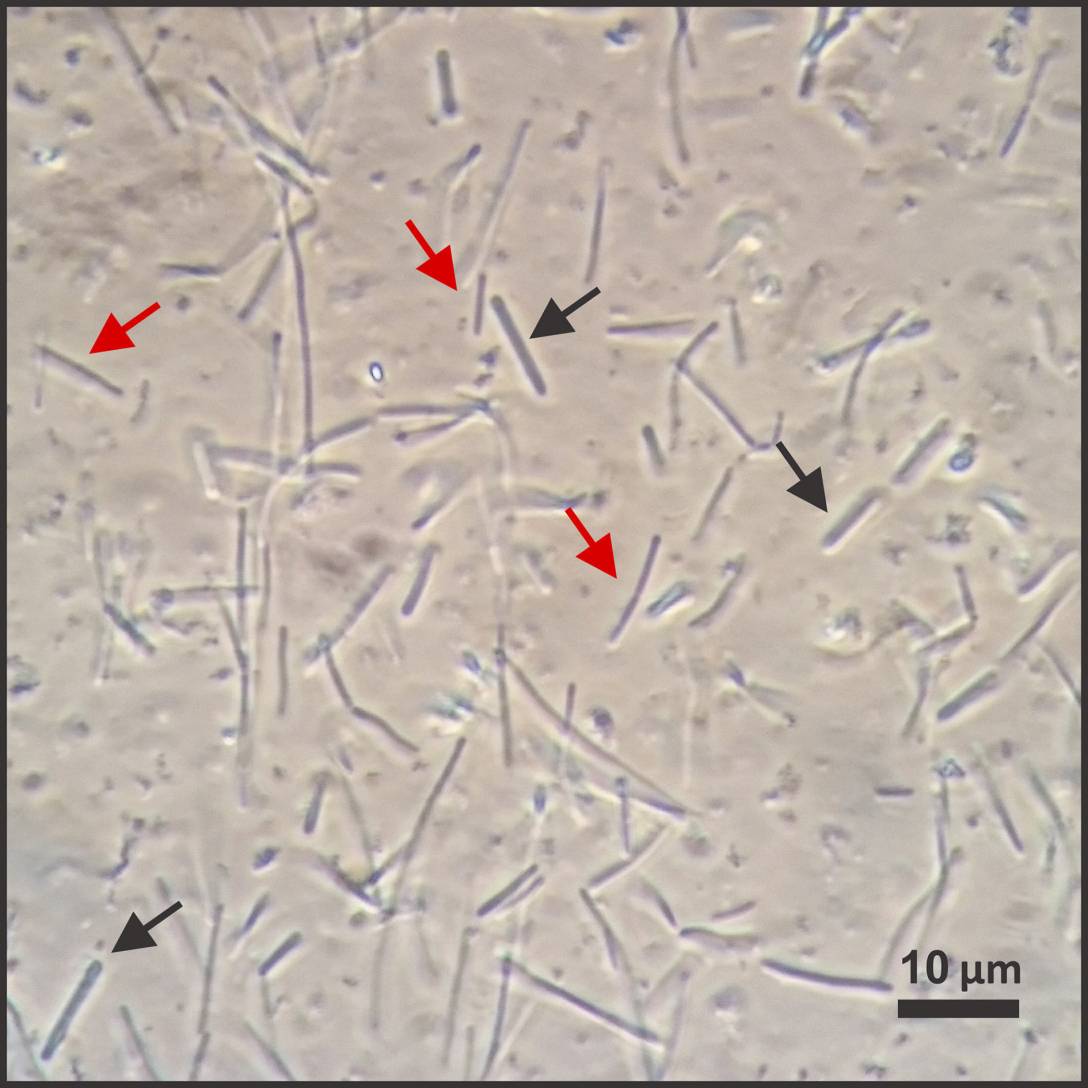
**Microscopy view of the co-culture with ***C. ljungdahlii*** and ***C. kluyveri*****. We used a phase-contrast microscope to periodically check the appearance of the co-culture. Black arrows point to cells of one morphology (thicker cells), while red arrows point to cells of another morphology (thinner cells). We observed this difference throughout the co-culture reactor run. However, it was not possible for us to clearly differentiate between *C. ljungdahlii* and *C. kluyveri* by light microscopy.

**Figure 4:**
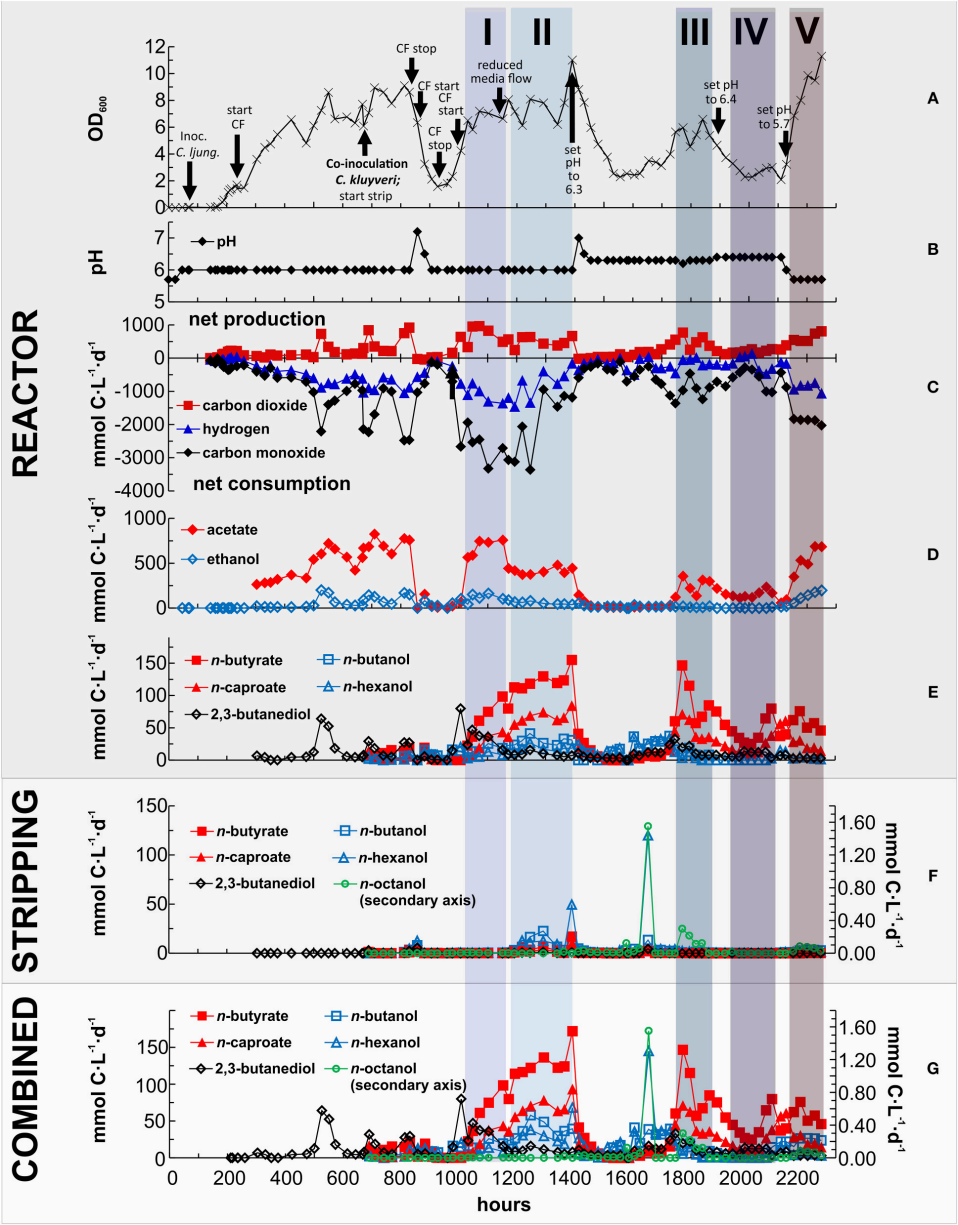
**Reactor performance during the continuous operation of ~2250 h (93 days)**. Data for one bioreactor run (*N* = 1 per Day) for **(A)** growth (OD_600_); **(B)** pH; **(C)** net gas consumption (negative) or production (positive) rates for CO_2_, H_2_, and CO in mmol C·L^−1^·d^−1^; **(D)** net production rates for acetate and ethanol in the reactor in mmol C·L^−1^·d^−1^; **(E)** net production rates for *n*-butyrate, *n*-caproate, 2,3-butanediol, *n*-butanol, and *n*-hexanol in the reactor in mmol C·L^−1^·d^−1^; **(F)** net production rates for *n*-butyrate, *n*-caproate, 2,3-butanediol, *n*-butanol, *n*-hexanol, and *n*-octanol in the stripping solution (condensate) in mmol C·L^−1^·d^−1^; **(G)** Combined net production rates (reactor + stripping solution) for *n*-butyrate, *n*-caproate, 2,3-butanediol, *n*-butanol, *n*-hexanol, and *n*-octanol in mmol C·L^−1^·d^−1^. The colored blocks labeled with I, II, III, IV, and V indicate the phases with stable reactor performance during continuous feeding, which we used for calculations. The high longer-chain alcohol production rates at hour 1650 were due to a clogging of the cell-recycling module, which led to an operation as fed-batch for ~12 h and caused an accumulation of 400 mL reactor broth in the condenser due to foaming. The foaming was indicative of a high metabolic activity. Together with the fed-batch operation this may explain the non-sustainable and only temporary accumulation of longer-chain products, which led to the high production rate of *n*-hexanol and *n*-octanol. This, however, was a non-sustainable experimental event. After solving the operational issue (i.e., clogging of the cell-recycling module) and switching back to continuous mode operation the accumulated products were washed out of the reactor and the steady-state production rates during Phase 3 were reached as described in the text. CF, continuous feed.

We operated a single-bioprocessing step with a co-culture for a period exceeding 2200 h (91 days; Figure 1). The reactor was continuously fed with a minimal nutrient broth solution and a procured syngas mixture of 60% CO, 35% H_2_, and 5% CO_2_. Periodic light microscopy monitoring revealed that the co-culture consisted of two distinct morphologies, which may be assigned to *C. ljungdahlii* (thinner rods) and *C. kluyveri* (thicker rods, Figure 3). However, this is speculative since other factors could have affected the morphology of the two microbes and we cannot reliably differentiate with light microscopy. Therefore, we also performed a non-quantitative PCR analysis with species-specific primers. We did not find DNA amplicons for *C. kluyveri* for three samples after inoculation with *C. kluyveri* when the chain elongation activity (i.e., production of longer-chain carboxylates) was low (Figure 2). However, we found amplicons for samples that were taken at hours 1250, 1430, and 1850 of the operating period when MCCs were measured in the reactor (Figure 2B). We observed resilience of the co-culture with a return to a dense cell population (OD_600_ between 5 and 10) after crashes in cell densities at hours 900 and 1600 (Figure 4A), which stemmed from an increase in the pH to 7 (Figure 4B). Even after these crashes, we observed chain elongation toward the end of the study, verifying that both *C. ljungdahlii* and *C. kluyveri* remained active in the co-culture. Together with the constant washout of 25% of bacterial cells in the effluent stream and the completely stirred conditions, we conclude that both strains showed sustainable growth and that they were resilient as a co-culture. Here, we operated one bioreactor and showed that the performance of the co-culture was stable during specific phases within the operating period. The results indicate that this process can be repeated. However, since we had several unexpected process perturbations, the exact profile might not be obtained again. Nevertheless, a well-functioning co-culture should be obtainable using the same process parameters.

**Figure 5:**
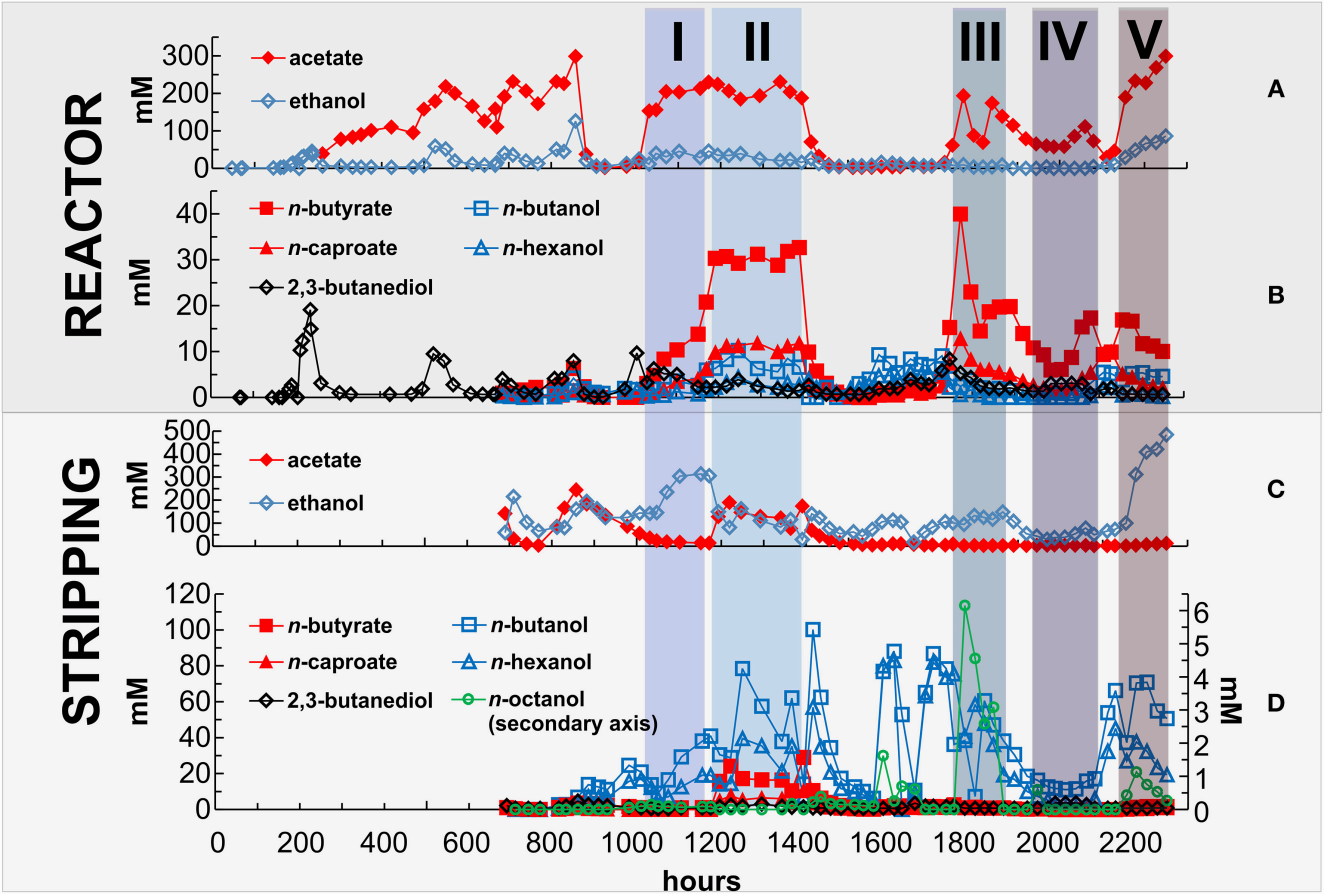
**Concentration of products in the reactor and in the stripping solution**. Data for one bioreactor run (*N* = 1 per Day) for **(A)** net acetate and ethanol concentrations in the reactor in mM; **(B)** net *n*-butyrate, *n*-caproate, 2,3-butanediol, *n*-butanol, and *n*-hexanol concentrations in the reactor in mM; **(C)** net acetate and ethanol concentrations in the stripping solution (condensate) in mM; **(D)** net *n*-butyrate, *n*-caproate, 2,3-butanediol, *n*-butanol, *n*-hexanol, and *n*-octanol concentrations in the stripping solution (condensate) in mM.

The co-culture operation was divided into five distinct phases with fully continuous operating conditions (Phase 1–5). During Phase 1 and 2, the pH in the reactor was 6.0 with either a high dilution rate (80 mL·h^−1^) or a low dilution rate (40 mL·h^−1^), respectively. During Phase 3, the pH was 6.3; during Phase 4, the pH was 6.4; and during Phase 5, the pH was 5.7; with low dilution rates of 40 mL h^−1^ during Phases 3–5 (Figure 4B). During all five phases, we observed promising CO and H_2_ consumption rates with simultaneous CO_2_ production rates (Figure 4C), and ethanol and acetate production rates (Figure 4D). We report the average net production rates (Figure 4) and concentrations (Figure 5) for the liquid chemical species during the five phases in the reactor effluent, in the condensate of the stripping system, and for the combined system (reactor, strip, and combined in Table 1). To report the production rates, we utilize mmol carbon (C) as the unit rather than just mmol to compare fairly within this study and between studies, and to not under-represent the production of longer-chain chemicals (1 mole of *n*-octanol is produced from at least 3 moles of ethanol (3xC2) plus 1 additional mole of ethanol or acetate (C2). Therefore, the volumetric production rates were expressed in mmol C per liter reactor volume per day (Table 1 and Figures 4C–G).

Table 1**Balances for substrates and products during phases of steady-state**.

**Table:** 

	**Phase**	**Substrates**	
		**CO**	**H_2_**
Consumption (mmol C·L^−1^·d^−1^)	1	2594 (500)	1106 (243)
	2	1899 (987)	766 (481)
	3	970 (313)	156 (170)
	4	603 (301)	158 (238)
	5	1893 (78)	881 (122)

**Table:** 

	**Phase**	**Products**									
		**CO**_2_	**EtOH**	**ButOH**	**HexOH**	**OctOH**	**2,3-BD**	**Ac**	**But**	**Capro**	**Capry**
Conc. Reactor	1	NA	31.2 (12.9)	2.05 (1.11)	0.64 (0.36)	<0.05	4.37 (1.62)	186 (29)	8.07 (4.31)	2.06 (1.63)	<0.04
(mM)	2	NA	27.7 (8.6)	7.39 (1.63)	2.93 (0.76)	<0.05	2.24 (0.85)	205 (18)	30.7 (1.38)	11.0 (0.8)	<0.04
	3	NA	6.06 (2.87)	2.44 (1.38)	0.835 (0.891)	0.138 (0.255)	4.06 (2.52)	120 (56)	21.8 (9.4)	7.72 (2.72)	<0.04
	4	NA	1.23 (2.11)	1.26 (0.86)	0.06 (0.15)	<0.05	2.23 (1.00)	73 (20)	10.5 (4.4)	2.8 (1.5)	<0.04
	5	NA	59.9 (21.7)	4.70 (0.62)	0.48 (0.23)	<0.05	0.69 (0.03)	244 (42)	13.3 (3.2)	3.4 (1.3)	<0.04
Conc. Strip	1	NA	228 (82)	21.3 (12.0)	11.4 (5.2)	0.10 (0.03)	0.55 (0.27)	22.2 (8.5)	0.68 (0.17)	ND	<0.04
(mM)	2	NA	105 (45)	44.2 (22.5)	23.9 (12.7)	0.0253 (0.0668)	1.87 (0.61)	139 (37)	18.4 (6.1)	6.42 (2.16)	<0.04
	3	NA	120 (20)	37.9 (17.6)	46.3 (19.4)	2.73 (2.45)	0.92 (0.49)	3.95 (2.61)	1.22 (0.69)	0.97 (0.36)	<0.04
	4	NA	47 (16)	13.7 (2.6)	3.6 (2.5)	0.08 (0.22)	2.21 (1.48)	3.31 (0.85)	0.05 (0.08)	0.33 (0.15)	<0.04
	5	NA	345 (150)	56.8 (14.2)	27.9 (7.2)	0.62 (0.33)	0.73 (0.09)	7.02 (4.40)	1.29 (0.48)	0.56 (0.40)	<0.04
Prod. rate reactor	1	710 (286)	114 (47)	14.9 (7.9)	6.96 (3.85)	ND	32.0 (12.3)	678 (94)	58.5 (30.2)	22.3 (17.4)	ND
(mmol C·L^−1^·d^−1^)	2	491 (157)	54.9 (14.7)	29.7 (6.3)	17.7 (4.6)	ND	8.99 (3.36)	412 (38)	124 (15)	67.1 (9.6)	ND
	3	508 (185)	12.2 (5.8)	10.3 (6.4)	5.34 (5.86)	ND	16.5 (10.1)	240 (97)	88.4 (35.6)	46.9 (15.9)	ND
	4	210 (45)	2.6 (4.5)	5.4 (4.1)	0.39 (1.02)	ND	9.27 (4.26)	152 (43)	44.2 (20.6)	17.9 (10.5)	ND
	5	623 (139)	136.0 (56.5)	20.9 (4.0)	3.16 (1.49)	ND	3.06 (0.27)	548 (143)	58.2 (11.6)	22.1 (6.8)	ND
Prod. rate strip	1	NA	1.94 (0.61)	0.035 (0.16)	0.32 (0.15)	0.0035 (0.0012)	0.0092 (0.0039)	0.198 (0.086)	0.0115 (0.0019)	ND	ND
(mmol C·L^−1^·d^−1^)	2	NA	10.6 (6.5)	9.49 (7.30)	14.0 (16.4)	0.0027 (0.0071)	0.487 (0.393)	18.7 (16.1)	5.29 (5.45)	2.83 (2.99)	ND
	3	NA	1.65 (0.76)	1.07 (0.70)	1.57 (1.34)	0.125 (0.118)	0.033 (0.043)	0.075 (0.104)	0.0447 (0.0584)	0.0493 (0.0532)	ND
	4	NA	0.32 (0.08)	0.19 (0.04)	0.08 (0.06)	0.0023 (0.0062)	0.0316 (0.0237)	0.024 (0.010)	0.0008 (0.0013)	0.0074 (0.0046)	ND
	5	NA	8.65 (4.94)	2.66 (0.96)	1.88 (0.50)	0.0557 (0.0270)	0.0345 (0.0127)	0.183 (0.136)	0.064 (0.034)	0.045 (0.037)	ND
Prod. rate comb	1	710 (286)	116 (48)	15.2 (8.0)	7.29 (4.00)	0.0035 (0.0012)	32.0 (12.3)	678 (93)	58.5 (30.2)	22.3 (17.4)	ND
(mmol C·L^−1^·d^−1^)	2	491 (157)	65.5 (18.3)	39.2 (10.8)	31.7 (17.8)	0.0027 (0.0071)	9.47 (3.46)	431 (44)	129 (20)	70.0 (12.3)	ND
	3	508 (185)	13.9 (5.8)	11.4 (6.4)	6.91 (6.96)	0.125 (0.118)	16.6 (10.1)	241 (97)	88.5 (35.6)	47.0 (15.9)	ND
	4	210 (45)	2.9 (4.5)	5.6 (4.1)	0.46 (1.03)	0.0023 (0.0062)	9.3 (4.3)	152 (43)	44.2 (20.6)	17.9 (10.5)	ND
	5	623 (139)	144.7 (61.4)	23.6 (4.8)	5.04 (1.82)	0.0557 (0.0270)	3.1 (0.3)	548 (143)	58.2 (11.6)	22.1 (6.8)	ND
% Selectivity	1	27.3 (10.5)	4.33 (1.40)	0.55 (0.21)	0.27 (0.10)	0.000144 (0.000073)	1.25 (0.48)	26.5 (3.7)	2.18 (0.98)	0.801 (0.571)	ND
(carbon-recovery)	2	32.1 (16.5)	3.99 (1.77)	2.57 (1.42)	2.16 (1.76)	0.000235 (0.000623)	0.58 (0.29)	28.5 (13.6)	8.71 (4.67)	4.73 (2.62)	ND
	3	53.1 (14.0)	1.55 (0.72)	1.46 (1.37)	0.90 (1.15)	0.0166 (0.0185)	1.95 (1.51)	27.8 (14.4)	10.9 (7.8)	5.78 (4.11)	ND
	4	42.6 (23.3)	0.46 (0.69)	0.96 (0.45)	0.052 (0.097)	0.000402 (0.001063)	2.05 (1.62)	28.4 (9.5)	7.5 (1.1)	2.95 (0.56)	ND
	5	32.8 (6.3)	7.58 (3.04)	1.25 (0.26)	0.27 (0.10)	0.00297 (0.00148)	0.16 (0.02)	28.8 (6.9)	3.1 (0.7)	1.18 (0.39)	ND
% Specificity	1	NA	12.5	1.64	0.78	0.000374	3.45	73.0	6.30	2.40	ND
(mol C/mol C)	2	NA	8.44	5.05	4.09	0.000346	1.22	55.5	16.7	9.01	ND
	3	NA	3.26	2.70	1.64	0.0298	3.90	57.2	21.0	11.2	ND
	4	NA	1.25	2.41	0.20	0.001	4.01	65.4	19.0	7.69	ND
	5	NA	18.0	2.93	0.63	0.0069	0.38	68.1	7.2	2.75	ND

*Relevant concentrations and rates are given, selectivities and specificities were calculated from these data (with standard deviations in brackets, where applicable). Averages and standard deviations were obtained by analysis of all daily samples for each phase*.

*Comb, reactor and stripping combined; Conc, concentration; Phase 1, high flow rate of growth medium (80 mL·h^−1^), pH 6.0, N = 5; Phase 2, low flow rate (40 mL·h^−1^), pH 6.0, N = 7; Phase 3, low flow rate (40 mL·h^−1^), pH 6.3, N = 6; Phase 4, low flow rate (40 mL·h^−1^), pH 6.4, N = 7; Phase 5, low flow rate (40 mL·h^−1^), pH 5.7, N = 5; mol C, mol carbon; prod, production; strip, stripping solution; % selectivity calculated as mol carbon in respective product compared to mol carbon in substrate (CO) consumed; % specificity calculated as mol carbon in specific product compared to mol carbon in all products (except CO_2_ which is not considered in this calculation). EtOH, ethanol; ButOH, n-butanol; HexOH, n-hexanol; OctOH, n-octanol; 2,3-BD, 2,3-butanediol; But, n-butyrate; Capro, n-caproate; Capry, n-caprylate*.

The highest average production rates for both longer-chain alcohols and MCCs occurred during Phase 2 (Table 1). During this phase, the co-culture produced the following eight liquid chemicals with the combined, net volumetric production rates in parentheses, ordered from high to low average rates: acetate (431 mmol C·L^−1^·d^−1^); *n*-butyrate (129 mmol C·L^−1^·d^−1^); *n*-caproate (70.0 mmol C·L^−1^·d^−1^); ethanol (65.5 mmol C·L^−1^·d^−1^); *n*-butanol (39.2 mmol C·L^−1^·d^−1^); *n*-hexanol (31.7 mmol C·L^−1^·d^−1^); 2,3-butanediol (9.47 mmol C·L^−1^·d^−1^); and *n*-octanol (0.045 mmol C·L^−1^·d^−1^) (*SD* in Table 1). During Phase 5, the highest average concentration of acetate (244 mM) was measured in the reactor (Figures 5A,C and Table 1), while the highest average concentrations of *n*-butyrate and *n*-caproate (30.7 and 11.0 mM, respectively) were found in the reactor during Phase 2 (Figures 5B,D and Table 1). Due to the concentrating effect of the gaseous stripping system for alcohols, the highest concentrations for ethanol, *n*-butanol, *n*-hexanol, and *n*-octanol (345, 56.8, 46.3, and 2.73 mM, respectively) were measured in the condensate compared to the reactor during different phases (Figures 5B,D and Table 1).

We achieved higher net production rates for all of these products in our continuous fermentation system compared to Diender et al. (2016) in their batch fermentation system with a different co-culture. Our production rates during Phase 2 were more than 2-fold higher for *n*-butanol and *n*-hexanol, more than 4-fold higher for *n*-caproate, and more than 9-fold higher for *n*-butyrate than theirs (Diender et al., 2016). As such, our system also achieved considerably higher production rates than with the reactor microbiome in a batch fermentation system of Ganigué et al. (2016). The chain elongation for these two studies had not observed longer-chain chemicals than C6. With the production of *n*-octanol this is, thus, the first study that observed C8 products within an anaerobic syngas fermentation system with wild-type strains. In the patent literature, a genetically modified CTB is described that can produce chemicals of up to C15 (farnesene) under anaerobic conditions (Chen et al., 2015). In addition, Hu et al. (2016) produced C16 and C18 products with genetically modified yeast, albeit under aerobic conditions in a separate bioprocessing step after the syngas fermentation step.

Between hour 1740 and 1860 of the operating period, we measured a concentration of up to 6 mM of *n*-octanol in the condensate of the gas stripping system (Figure 5D), which is the highest concentration we measured. This resulted in a net average production rate of 0.125 mmol C·L^−1^·d^−1^ for *n*-octanol (Figure 4G), and occurred during Phase 3 at a reactor pH of 6.3, which stimulated *C. kluyveri* to chain elongate with available ethanol and carboxylates. The higher pH of 6.3 compared to 6.0 is advantageous for *C. kluyveri* due to two reasons: (1) the growth conditions are more optimum (Barker and Taha, 1942); and (2) the concentrations of undissociated medium-chain carboxylic acids are lower (pKa of ~4.9), resulting in a lower microbial inhibition (Butkus et al., 2011). The resulting *n*-octanol production was achieved at a low selectivity and specificity (mol carbon in product per mol carbon in all other liquid products). During Phase 3, the selectivity and specificity for *n*-octanol was ~0.02 and 0.03%, respectively (Table 1). The production rate for *n*-octanol did not further increase when we increased the pH to 6.4 during Phase 4, most likely because of the lower *C. ljungdahlii* activity that provided less ethanol and acetate, decreasing the overall productivity of the process. We were able to observe the low production rates for *n*-octanol due to the accumulation effect in the condensate of the gaseous stripping system. The *n*-octanol concentrations in the reactor effluent remained below the detection limit for our GC method (0.01 mM). In addition, the concentrations of *n*-caprylate in the reactor effluent and condensate remained below the detection limit (0.04 mM).

### A narrow, but not ideal, pH spectrum available for the production of longer-chain alcohols

During Phase 2 at a pH of 6.0, the specificities (calculated on the basis of the production rates in mmol C·L^−1^·d^−1^) for ethanol, 2,3-butanediol, and acetate were 8.44, 1.22, and 55.5%, respectively, with a total of ~65% for the products from wild-type *C. ljungdahlii* (Table 1). Meanwhile, the specificities for *n*-butanol, *n*-hexanol, *n*-octanol, *n*-butyrate, and *n*-caproate were 5.05, 4.09, <1, 16.7, and 9.01%, respectively (Table 1), with a total of ~35% for the longer-chain chemicals after chain elongation with wild-type *C. kluyveri*. A simplified ratio of activity for *C. ljungdahlii* compared to *C. kluyveri* would be 1.9 (65/35) when the biological reduction of MCCs is not included in the activity for *C. ljungdahlii*. However, this ratio is based on the net production rates, and because ethanol and acetate are removed during chain elongation, we would underestimate the importance of *C. ljungdahlii*. Next, we used the approach by Diender et al. (2016) to estimate the total volumetric production rates by including the production of intermediates (Table 2). Compared to the net volumetric production rates, the total volumetric production rates increased the ratio of activity for *C. ljungdahlii* compared to *C. kluyveri* from 1.9 to 2.2 [(196 + 554)/(236 + 102) in Table 2]. We also estimated that 2/3 [(196 − 65.5)/196 in Tables 1, 2] of the total ethanol production was used to chain elongate with *C. kluyveri*.

**Table 2:** **Total production rates in mmol carbon (C)·L^**−1**^·d^**−1**^ for acetate, ethanol, ***n***-butyrate, and ***n***-caproate (with standard deviations in brackets), calculated as the sum of combined production rates (see Table 1) of carbon chains that require each of the respective product as precursor**.

	**Phase**	**Total production rate (mmol C·L^−1^·d^−1^)**
**Acetate_total_**	1	812 (129)
	2	554 (43)
	3	284 (99)
	4	168 (46)
	5	715 (201)
**Ethanol_total_**	1	167 (67)
	2	196 (25)
	3	95 (31)
	4	40 (22)
	5	200 (56)
***n*****-Butyrate_total_**	1	93 (50)
	2	236 (43)
	3	136 (49)
	4	62 (32)
	5	100 (17)
***n*****-Caproate_total_**	1	30 (21)
	2	102 (29)
	3	54 (20)
	4	18 (11)
	5	27 (7)

*Acetate_total_* = *acetate_Comb_* + *ethanol_comb_* + *1/2* × *n-butyrate_comb_* + *1/2* × *n-butanol_comb_* + *1/3* × *n-caproate_comb_* + *1/3* × *n-hexanol_comb_* + *1/4* × *n-octanol_comb_*.

*Ethanol_total_* = *ethanol_comb_* + *1/2* × *n-butyrate_comb_* + *1/2* × *n-butanol_comb_* + *2/3* × *n-caproate_comb_* + *2/3* × *n-hexanol_comb_* + *3/4* × *n-octanol_comb._*

*n-Butyrate_total_* = *n-butyrate_comb_* + *n-butanol_comb_* + *2/3* × *n-caproate_comb_* + *2/3* × *n-hexanol_comb_* + *1/2* × *n-octanol_comb._*

*n-Caproate_total_* = *n-caproate_comb_* + *n-hexanol_comb_* + *3/4* × *n-octanol_comb_*

*Acetate_total_ is calculated as the maximum possible rate assuming the highest value of 1 mol acetate consumed for production of each mol of ethanol, n-butyrate, n-butanol, n-caproate, n-hexanol, and n-octanol. The real value is between 0 and 1 mol acetate for each mol of medium-chain product, and therefore the actual values for acetate_total_ are likely lower than calculated here*.

*Ethanol_total_ is calculated as the minimum required rate assuming 1, 2, and 3 mol ethanol required per mol of 4-carbon, 6-carbon, and 8-carbon product, respectively. The actual values for ethanol_total_ are likely higher than calculated here*.

During the operating period, we attempted to optimize the conditions for production of longer-chain alcohols and MCCs. Initially, we had started the operating conditions of the reactor at a pH of 6.0. Based on the studies by Diender et al. (2016) and Ganigué et al. (2016), we knew that a discrepancy exists in optimum pH levels for ethanol production by CTB and growth by *C. kluyveri*. Since, ethanol production is a prerequisite for chain elongation by *C. kluyveri*, we set the pH at 6.0. During the startup phase with only *C. ljungdahlii*, we observed ethanol production rates of up to 170 mmol C·L^−1^·d^−1^, but with acetate as the main product (721 mmol C·L^−1^·d^−1^) and some 2,3-butanediol (52 mmol C·L^−1^·d^−1^; Figures 4D,E). We inoculated *C. kluyveri* on hour 670 at this pH of 6.0 and observed some production of *n*-butanol, *n*-hexanol, *n*-butyrate, and *n*-caproate in addition to similar ethanol and acetate production rates (Figures 4D,E). From this, we concluded that we had established a co-culture of *C. ljungdahlii* and *C. kluyveri*, however, chain elongation was not very active. For that, the co-culture had to go through a pH perturbation.

Two operating problems at hour 850 and 1400 showed us that a pH of 7.0 or higher would have been a bad choice for sustainable chain elongation in the co-culture. First, a malfunction of the peristaltic pump for the continuous medium flow at hour 850 changed the reactor from a continuous to a batch system (Figure 4A). Because of the lack of an acid pumping system to control the upper pH limit, we observed an uncontrolled increase in the pH from 6.0 to 7.2. A similar pH increase had been observed by Diender et al. (2016) in batch fermentations with a co-culture of *C. autoethanogenum* and *C. kluyveri*. This neutral pH crashed the population of *C. ljungdahlii* because they cannot gain ATP via RNF complex/ATP synthase with a dissipated proton gradient, and thus cannot grow (Tanner et al., 1993). Without ethanol production from *C. ljungdahlii*, we observed an imminent collapse of the co-culture (Figures 4C–F). After a period of batch conditions at a pH of 6.0, the *C. ljungdahlii* population recovered (Figure 4A), with sufficient syngas consumption (Figure 4C) after which the continuous operating conditions resumed (Phase 1). Second, a controlled pH increase to 6.3 at hour 1400 stimulated chain elongation so much that the pH again increased uncontrollably, resulting in the same negative outcome (Figures 4C–F).

For unknown reasons to us, however, the first pH perturbation seem to have given *C. kluyveri* an enduring advantage, resulting in considerably higher net production rates of longer-chain chemicals during Phase 1 at a pH of 6.0 (Figures 4E,G). After steady-state acetate production rates had been established during Phase 1 at a continuous medium flow rate of 80 mL·h^−1^, we reduced the flow rate to 40·mL h^−1^ during Phase 2. This change in flow rate increased the average concentration of acetate from 186 to 205 mM (Table 1), which we anticipated would increase solventogenesis in *C. ljungdahlii* (Richter et al., 2016), with a lower 2,3-butanediol production rate (Figure 4E). Even though this change resulted in lower net ethanol and acetate production rates (Figure 4D), the net production rates for all other observed products increased (Figure 4G). This means, that under these conditions the net activity for *C. kluyveri* was higher than during Phase 1, resulting in the highest observed chain elongation rates in this study.

Because only 2/3 of the produced ethanol was removed during Phase 2 (Figure 4D), we concluded that the activity by *C. ljungdahlii* was not the rate limiting step in the co-culture at a pH of 6.0. Therefore, we increased the pH to 6.3 to boost the activity by *C. kluyveri*, while we anticipated that a further reduction in the activity by *C. ljungdahlii* was tolerable. Before increasing the pH during Phase 3, we installed an upper pH control with acetic acid to maintain a pH of 6.3 and to prevent a third pH perturbation. On average ~0.3 mL·d^−1^ acetic acid was pumped after installing the upper pH control. This corresponds to only 0.6 mM·d^−1^ acetic acid, and therefore was negligible in our calculations. We observed a stabilization of the co-culture with production rates slightly lower than during Phase 2. As we had anticipated, the specificity for the net production of ethanol was reduced from 8.44 to 3.29%, while the specificity for acetate, *n*-butyrate, and *n*-caproate was slightly increased compared to Phase 2 (Table 1). Unfortunately, the biological reduction of MCCs to longer-chain alcohols by *C. ljungdahlii* was also slowed due to the higher pH of 6.3. This resulted in a lower specificity of the longer-chain alcohols except for *n*-octanol (Table 1). We postulate that the decrease in biological reduction activity may be due to several reasons: (1) the growth of *C. ljungdahlii* is slower; (2) the concentrations of the undissociated medium-chain carboxylic acids, which are the substrates for the biological reduction, are lower (e.g., 3% at pH 6.3 compared to 6% at pH 6.0 for acetic acid) (Richter et al., 2016); and (3) the lower CO-uptake by *C. ljungdahlii* may have increased the dissolved CO concentrations, reducing the *C. kluyveri* activity (Diender et al., 2016). However, the slightly higher pH of 6.3 compared to 6.0 increased the combined, net average production of *n*-octanol from 0.003 to 0.125 mmol C·L^−1^·d^−1^ (Figures 4F,G), resulting in a ~10-fold increase in the specificity for *n*-octanol to 0.03% during Phase 3 (Table 1).

We did find an increase in the relative chain elongation activity at the higher pH of 6.3. Besides the increased net production rates of the MCCs, a low net H_2_ consumption rate due to H_2_ production from chain elongation by *C. kluyveri* was also indicative of this increased activity in chain elongation (Figure 4C). Even though the net production rates of longer-chain alcohols during Phase 3 were not as high as during Phase 2, the slightly higher pH of 6.3 compared to 6.0, reduced the relative concentration of undissociated medium-chain carboxylic acids, which represent the volatile species, compared to dissociated MCCs. This resulted in a lower loss of carboxylate product from the reactor broth and a cleaner condensate with almost only higher-chain alcohols (Figure 5D). We did not measure esters (derived from a carboxylic acid and an alcohol) in the condensate, but we did observe a fruity smell of pineapple.

A further increase in the pH to 6.4 during Phase 4 was in agreement with the above finding for Phase 3. The specificity for the net production rate of ethanol was further reduced from 3.26 to 1.25% (Table 1). In addition, the low ethanol availability likely decreased chain-elongation activity by *C. kluyveri*, which led to lower specificities for the net production rates of the medium-chain carboxylic acids (Table 1). This and further reduced biological reduction activity also decreased the specificities for the longer-chain alcohols (Table 1). Therefore, at a pH of 6.4 the low *C. ljungdahlii* activity is limiting the overall productivity of the process. To investigate the lower pH limit of the process, we decreased the pH again to 5.7 during Phase 5. At that pH we found the highest net ethanol production rate reported in this study (144.7 mmol C·L^−1^·d^−1^) and the second highest net acetate production rate (548 mmol C·L^−1^·d^−1^; Table 1). The specificity for the production rates of the longer-chain alcohols was comparable to Phase 3 and 4 (Table 1). However, in this case not the low biological reduction activity by *C. ljungdahlii* was limiting, but the reduced chain-elongation activity by *C. kluyveri*, which led to low production rates of MCCs (Table 1).

## Discussion

### A continuous co-culture bioprocessing step shows resilience over a long operating period

In this study, we have considerably enhanced the proof-of-concept by Diender et al. (2016) to combine syngas fermentation, chain elongation, and biological reduction into one bioprocessing step with a defined co-culture of wild-type strains. Our bioprocessing system was continuously fed rather than operated as a batch, and continuous product extraction was included. Both wild-type strains in our co-culture remained active and showed resilience during an operating period of more than 1500 h (62 days). The result is a bioprocess that can recover C1 compounds into much higher value C4–C8 chemicals, including longer-chain alcohols such as *n*-hexanol and *n*-octanol. Of the small number of studied pH-values, the optimum reactor pH was found to be 6.0 during which the production rates for either *n*-butanol and *n*-hexanol were ~30–40 mmol C·L^−1^·d^−1^, but at a relatively low specificity of ~4–5% due the co-production of ethanol, 2,3-butanediol, acetate (main product), *n*-butyrate, *n*-caproate, and traces of *n*-octanol. With this, we verified a previously described pH discrepancy between currently available CTB and chain-elongating bacteria (Diender et al., 2016; Ganigué et al., 2016).

### Product specificity may remain low even after optimization

Therefore, regardless of what the best pH is for the maximum net production rates of longer-chain alcohols, the observed pH discrepancy for optimum function between *C. ljundahlii* and *C. kluyveri* makes this a less promising biotechnology production platform at this point in time. It is imperative to isolate a different chain-elongation bacterial strain with a growth optimum that is between 5 and 5.5. The operating performance at the lower pH would increase the total ethanol production rates by *C. ljungdahlii*. The matching pH optimum would then also lead to more equal activities of ethanol production and chain elongation, possibly yielding the promising production rates of longer-chain alcohols that are necessary. A high production rate of such alcohols can only occur with a gaseous stripping system or a different product extraction system that maintains the concentration of these alcohols low enough to prevent product inhibition. This stripping system may need to be different from the current system when the goal is to mainly extract longer-chain alcohols during a lower operating pH in the reactor.

But even when we acquire a co-culture with matching pH optima and install an ideal product extraction system, the specificities for the longer-chain alcohols may still remain low. This due to the competition for carboxylates as substrates between chain elongation and biological reduction in the single bioprocessing step that leads to a mixture of several longer-chain alcohols. In a system that performs exclusively chain elongation, the specificity for one single product can be much improved. For example, Agler et al. achieved 79% specificity for *n*-caproic acid (Agler et al., 2012). With chain elongation and biological reduction competing, we and others have observed mixtures of alcohols (Diender et al., 2016; Ganigué et al., 2016). The resulting low specificities raises the question whether we can ever achieve a truly promising chemical production platform within a single bioprocessing step for syngas fermentation with defined co-cultures of wild-type bacterial strains? On the other hand, a mixture of longer-chain alcohols may be suitable for certain application areas such as in the biofuel industry. In addition, an additional product separation step, such as distillation, after the stripping system could further separate the different alcohols.

### Modeling and comparison of different strategies for bioprocessing steps necessary

Alternatively, the combination of separate bioprocessing steps with a pure culture of CTB and chain-elongating bacteria in either mixed or pure cultures would allow for a more precise control. A syngas fermentation bioprocess as the first step (ethanol production), coupled to a chain-elongation bioprocess as the second step (*n*-caproate production), and another syngas fermentation bioprocess as the third step (*n*-hexanol production)—all three bioprocessing steps in separate reactors—may result in a considerably higher specificity for alcohols. Another possibility is to couple two bioprocessing steps within a recycle loop to perform ethanol production and biological reduction of MCCs in the same bioprocessing step with syngas.

A comparison of the three strategies (single bioprocessing step, three separate bioprocessing steps, two separate bioprocessing steps combined as a recycling loop) by modeling of the product spectrum under different process conditions could be used to get a better understanding. Here, we provided a proof-of-concept for the resilience and functionality of a single bioprocessing step with a co-culture of pure strains in continuous operation mode. Which of the three strategies will eventually be the best choice as a bioproduction platform at an industrial scale cannot be answered at this point in time and more applied research is necessary. The choice may also be depending on whether a pure product or a mixture of long-chain alcohols is desired.

## Author contributions

HR, BM, MD, DS, and LA designed the study; HR and BM performed the research and sample analysis; HR and BM analyzed the data, and HR, BM, MD, DS, and LA wrote the manuscript.

## Funding

This work was supported by the NSF SusChEM Program (Award #1336186) and by the U.S. Army Research Laboratory and the U.S. Army Research Office (Contract/Grant number W911NF-12-1-0555), which were awarded to LA. In addition, BM was funded through a postdoctoral research fellowship from the German Research Foundation (DFG, MO2933/1-1). Research of MD and DS is financed by the European Research Council (ERC Grant Agreement 323009) and the Netherlands Ministry of Education, Culture and Science (Gravitation grant 024.002.002).

### Conflict of interest statement

The authors declare that the research was conducted in the absence of any commercial or financial relationships that could be construed as a potential conflict of interest.
